# The complete mitochondrial genome of the jewel beetle *Trachys variolaris* (Coleoptera: Buprestidae)

**DOI:** 10.1080/23802359.2019.1666053

**Published:** 2019-09-18

**Authors:** Liangming Cao, Xiaoyi Wang

**Affiliations:** The Key Laboratory of Forest Protection, State Forestry and Grassland Administration of China, Research Institute of Forest Ecology, Environment and Protection, Chinese Academy of Forestry, Beijing, China

**Keywords:** Mitochondrial genome, Elateriformia, Buprestidae, *Trachys variolaris*

## Abstract

The complete mitochondrial genome (mitogenome) of the jewel beetle, *Trachys variolaris*, was sequenced and described. The mitogenome of *T. variolaris* is a typical circular DNA molecule with 16,771 bp in length. It contains the typical 37 mitochondrial genes (13 protein-coding genes, two rRNA genes, and 22 tRNA genes) and a long non-coding region called the control region. Twelve protein-coding genes initiate with ATN codons except *ND1* uses TTG. Most of the protein-coding genes use TAA or TAG as the stop codon, but *COII*, *COIII*, *ND4*, and *ND5* terminate with a single T–– or TA–. The length of the 22 tRNAs ranges from 61 to 70 bp and they all have the clover-leaf structure except for *tRNA^Ser(AGN)^*. The control region is 2155 bp long with the A + T content of 69.4%. The result of our phylogenetic analysis showed that Buprestoidea is monophyletic, and it is the sister group to (Byrrhoidea + Elateroidea).

Jewel beetles (Buprestidae) are famous for their beautiful metallic sheen but most of them are important forestry pests as their larvae are either xylophagous and wood-boring, or stem- and leaf-mining. Generally, the bodies of Buprestidae are elongated or even narrow and cylindrical, but species in the genus *Trachys* possess a short body with a round or drop-shaped form (Bernhard et al. 2005). Among them, *Trachys variolaris* was firstly described by Saunders in 1873 (Saunders 1873) and can be collected from *Quercus* trees. Here, we sequenced and described the complete mitogenome of the jewel beetle, *T. variolaris*. Voucher specimen (No. VCim-00112) was deposited at the Entomological Museum of Chinese Academy of Forestry and the sequence was submitted to GenBank under the accession number MN178497.

The complete mitochondrial genome of *T. variolaris* is a typical circular DNA with 16,771 bp in length, containing 37 genes (13 protein-coding genes, 22 tRNA genes, and two rRNA genes) and a long non-coding region. The arrangement of genes is identical with the gene order of *Drosophila yakuba*, which is regarded as the putative ancestral one (Clary and Wolstenholme 1985; Cameron 2014). Totally, there are 51 bp overlapped nucleotides between adjacent genes in 17 locations, ranging from 1 to 8 bp in size. Except for control region, seven inter-genic regions were found in this mitogenome, ranging from 1 bp to 25 bp.

The nucleotide composition of the whole mitogenome is significantly biased toward A and T. The A + T content is 72.1% with positive AT-skew (0.11) and negative GC-skew (−0.21). All protein-coding genes initiate with ATN codons except for *ND1* uses TTG. The stop codon TAA and TAG are assigned to nine protein-coding genes, whereas *COII*, *COIII*, *ND4*, and *ND5* terminate with TA– or a single T–– residue. Using a single T–– or TA– as stop codon is commonly found in many other insect mitogenomes (Hong et al. 2009; Zhao et al. 2019; Cao and Wang 2019).

This mitogenome contains all set of typical 22 tRNA genes and the length ranges from 61 bp to 70 bp. Among them, only *tRNA^Ser(AGN)^* cannot be folded into the clover-leaf secondary structure as the result of the deficiency of the dihydrouridine (DHU) arm. The *tRNA^Ser(AGN)^* with a simply looped DHU arm is also found in many insects (Li et al. 2012; Song et al. 2016). The *lrRNA* is 1320 bp long with a high A + T content (79.3%), and the *srRNA* is 791 bp long with an A + T content of 77.2%. The control region is located between *srRNA* and *tRNA^Ile^* with 2155 bp in length.

Phylogenetic tree of Elateriformia based on the dataset of 13 PCGs was conducted by maximum-likelihood (ML) method (Figure 1). The results confirmed that the four superfamilies within Elateriformia are all monophyly. Buprestoidea is shown as the sister group to the clade (Byrrhoidea + Elateroidea). These results are congruent with previous hypotheses on the phylogenetic relationships among Elateriformia using molecular data (Bocakova et al. 2007; Kundrata et al. 2017).

**Figure 1.**
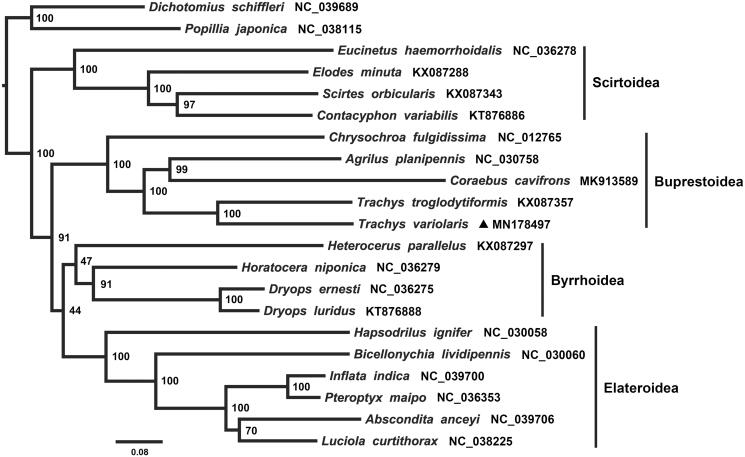
Phylogenetic relationship of 19 species among Elateriformia. The phylogenetic tree was conducted by ML analysis of the 13 protein-coding genes (10,992 bp) with IQ-TREE 1.6.5 (Trifinopoulos et al. 2016). The nodal values indicate the bootstrap percentages obtained with 10,000 replicates. Alphanumeric terms indicate the GenBank accession numbers.
